# Effects of Meat Cooking, and of Ingested Amount, on Protein Digestion Speed and Entry of Residual Proteins into the Colon: A Study in Minipigs

**DOI:** 10.1371/journal.pone.0061252

**Published:** 2013-04-12

**Authors:** Marie-Laure Bax, Caroline Buffière, Noureddine Hafnaoui, Claire Gaudichon, Isabelle Savary-Auzeloux, Dominique Dardevet, Véronique Santé-Lhoutellier, Didier Rémond

**Affiliations:** 1 Clermont Université, Université d'Auvergne, Unité de Nutrition Humaine, Clermont-Ferrand, France; 2 INRA, UMR1019 UNH, CRNH Auvergne, Clermont-Ferrand, France; 3 INRA, UR370 Qualité des Produits Animaux, Saint Genès Champanelle, France; 4 INRA-AgroParisTech, CRNH Ile de France, UMR914 Nutrition Physiology and Ingestive Behavior, Paris, France; National Institute of Agronomic Research, France

## Abstract

The speed of protein digestion impacts on postprandial protein anabolism. After exercise or in the elderly, fast proteins stimulate protein synthesis more efficiently than slow proteins. It has been shown that meat might be a source of fast proteins. However, cooking temperature, acting on the macrostructure and microstructure of the meat could affect both the speed, and efficiency, of protein digestion. This study aims to evaluate, *in vivo*, the effect of meat cooking on digestion parameters, in the context of a complete meal. Six minipigs fitted with an ileal cannula and an arterial catheter were used. In order to measure the true ileal digestibility, tested meat was obtained from a calf, the muscle proteins of which were intrinsically labelled with ^15^N-amino acids. Three cooking temperatures (60, 75 and 95°C; core temperature for 30 min), and three levels of intake (1, 1.45, and 1.90 g protein/kg body weight) were tested. Following meat ingestion, ileal digesta and arterial blood were collected over a 9-h period. The speed of digestion, evaluated from the kinetics of amino acid appearance in blood within the first 3 h, was greater for the cooking temperature of 75°C, than for 60 or 95°C. The true ileal digestibility, which averaged 95%, was not affected by cooking temperature or by the level of meat intake. The amino acid composition of the digesta flowing at the ileum was not affected by cooking temperature. These results show that cooking temperature can modulate the speed of meat protein digestion, without affecting the efficiency of the small intestinal digestion, and consequently the entry of meat protein residues into the colon.

## Introduction

The classic criteria for evaluating the quality of a protein source are based on amino acid (AA) composition and digestibility of the protein fraction along the length of the digestive tract. It is now known that these basic criteria are not sufficient to fully describe the nutritional potential of a protein. For instance, it has been shown that the speed of protein digestion regulates postprandial protein retention [1]. Also, total digestibility is not a good predictor of amino acid bioavailability; indeed, only small intestinal digestion is thought to supply amino acids to the body. Measurements of true ileal digestibility (TID) are therefore more appropriate, but they are very difficult to obtain in healthy humans. Measurement of TID has been carried out on isolated proteins, by collection of ileal chyme via naso-intestinal tube [2]–[6], but there are no data on the TID of proteins in their natural matrix.

Meat proteins have a favourable balance of indispensable amino acids (IAA) and high digestibility in the whole digestive tract [7], [8]. They are very efficient at stimulating muscle protein synthesis in young and elderly subjects [9], and can be a significant source of bioactive peptides, such as carnosine [10], [11] and antihypertensive peptides [12]. Conversely, epidemiological studies have reported a possible link between excessive red meat intake and colorectal cancer risk [13], [14]. This relationship is, however, regularly questioned [15], [16] and moderate lean red meat consumption as part of a balanced diet is unlikely to increase cancer risk.

Regarding digestion, meat seems to be a source of fast-digested proteins [17], with a high TID in humans [18]. However, data on meat protein digestion are scarce, and do not enable the effect of the different processes involved in meat preparation to be taken into account. Yet, *in vitro* data have suggested that, for example, cooking temperature may affect the speed and efficiency of protein digestion [19]. As meat protein degradation in the colon has been suspected as a potential cause of cancer development, a better knowledge of meat protein digestibility in the small intestine is required.

In this context, the present study aimed to investigate, *in vivo*, the effect of meat cooking temperature on the parameters of protein digestion in the small intestine, using the minipig as a model animal. Veal was used as the model meat, with three cooking temperatures and three levels of intake. For the determination of the TID coefficient, requiring the measurement of ileal dietary protein flow (versus endogenous protein flow), an original approach was used, involving ^15^N-amino acid incorporation in muscles before calf slaughter and meat preparation.

## Materials and Methods

All procedures were conducted in accordance with the guidelines formulated by the European Community for the use of experimental animals (L358-86/609/EEC), and the study was approved by the Local Committee for Ethics in Animal Experimentation (N°CE9-11; Comité d'Ethique en Matière d'Expérimentation Animale d'Auvergne, Aubière, France). All surgeries were performed under gaseous general anaesthesia, using isoflurane, and all efforts were made to minimize suffering.

### Animals

The study involved six female Göttingen minipigs (Ellegaard, Denmark) (12–16 mo old; 20–25 kg body weight). At least 3 weeks before initiating the experiment, minipigs were surgically fitted with a permanent catheter (polyvinyl chloride; 1.1-mm i.d., 1.9-mm o.d.) in the aorta, and a T-shaped cannula (silicone rubber; 12-mm i.d., 17-mm o.d.) in the distal ileum. Surgical procedures, as well as post-surgical care, have been previously described in detail by Rémond et al. [20]. Minipigs were housed in individual pens (1×1.5 m), separated by Plexiglass® walls, in a ventilated room with controlled temperature (20–23°C). They were fed once daily, at 0815, with 400 g of a commercial feed [18% protein (nitrogen (N) ×6.25), 2% fat, 5% cellulose, 6% ash] (Porcyprima, Sanders Nutrition Animale, France), and had free access to water.

### Meat

A calf (Montbelliard X Charolais; 86 kg body weight) was fitted with a catheter in the jugular vein. This catheter was used to infuse continuously, over a 14-d period, a solution containing a mixture of ^15^N-amino acids (algal amino acids, 98% ^15^N, Cambridge Isotope Labs. Inc., MA). The solution was made up with 26 g of the ^15^N-amino acid mixture, diluted in 1 l of sterile apyrogenic water (pH 7.4; 330 mOsmole/l), and autoclaved. It was infused at a rate of 4 ml/h, using a syringe pump (Vial medical, SE 400, Becton Dickinson, Germany). The calf was slaughtered 1 d after the end of the infusion in order to ensure a decrease in ^15^N-enrichment of muscle free amino acids. The carcass was kept for 5 days at 4°C. The *longissimus dorsi* muscle was sampled for experiment 1 (exp. 1) and the *semimembranosus* and *biceps femoris* muscles were sampled for experiment 2 (exp. 2).

### Test meals

Muscles were cut into slices (1 cm thick). Each slice was weighed, bagged, and cooked in a water bath for 30 minutes at the selected core temperature (Exp. 1: 60, 75 or 95°C; Exp. 2: 75°C). Slices were then rapidly cooled in ice and reweighed to calculate juice loss during cooking. The cooked meat was minced (8-mm grid) in order to limit the effect of individual chewing efficiency of the minipigs. After mincing, meals were prepared by adding to the labelled meat: 100 g of wheat starch, 7 g of cellulose, 70 ml of water, and 25 g of fat. Furthermore, 1 g of the undigestible marker chromic oxide (Cr_2_O_3_) was added, to correct for losses of chyme not exported through the cannula [21]. After mixing the ingredients, the meal was presented as balls of 1.5–2.0 cm in diameter, to facilitate ingestion by the minipigs. Meals were placed under partial vacuum and stored at −20°C until use.

### Experimental protocol

The study included two experiments. Exp. 1 investigated the effect of cooking temperature (60, 75, or 95°C) on small intestinal digestibility and the kinetics of amino acid absorption. In this experiment, the amount of meat in the different test meals was adjusted in order to take into account the juice losses during cooking, and to provide the same amount of protein in each test meal (30 g). Exp. 2 investigated the effect of the amount of ingested meat (65, 100, or 135 g of meat cooked at 75°C, corresponding to 1, 1.45, and 1.90 g protein/kg body weight, respectively) on small intestinal digestibility. In each experiment, all test meals were tested on all six minipigs, according to a duplicate 3×3 latin square design. For a given minipig, sampling days were separated by at least one week. On days in between the sampling days, minipigs received the commercial feed. On the day of sampling, minipigs did not receive the commercial feed but were exclusively offered the test meals (at 0815), and given continuous access to water. Digesta were continuously collected from 30 min before to 9 h after test meal delivery. Digesta were collected 30 min before the meal in order to measure basal ^15^N enrichment. Plastic bottles, attached to the cannula, were regularly replaced according to digestive burst. The collected digesta (accumulated over 1-h intervals) were weighed and immediately frozen at −20°C. During exp. 1, blood samples (5.5 ml) were collected in cold syringes with lithium-heparin as an anticoagulant (S-Monovettes, Starstedt), at 0745, 0830, 0845, 0915, 0945, 1045, 1145, 1245, 1415 and 1545. They were immediately centrifuged at 1,500 g for 10 min at 4°C. The resulting supernatant was frozen in liquid nitrogen and stored at −80°C.

### Analytical methods

Digesta were lyophilised, and homogenised using a ball mill. For chromium determination, freeze-dried samples were mineralised at 550°C for 6 h. Ashes were dissolved in a mixture of nitric acid and chromium, and concentration was determined using atomic absorption spectrometry (AAnalyst 400, Perkin-Elmer). Total nitrogen levels in the gastrointestinal effluents and meat were determined using an elemental analyser (Vario Isotope cube, Elementar). For amino acid analysis, a representative sample of the whole postprandial period was constituted from a fixed percentage of each hourly-collected digesta. Prior to acid hydrolysis (HCl 6N, 24 h, 110°C), sulphur-containing amino acids were oxidised with H_2_O_2_. This pre-treatment allows determination of sulphur-containing amino acids but leads to an underestimation of threonine and tyrosine residues, which are particularly sensitive to oxidation. Amino acids were determined by ion exchange chromatography, with ninhydrin post-column reaction (Bio-Tek Instruments). Determination of ^15^N enrichment was performed using an isotope ratio mass spectrometer (Optima, Fisons Instruments) coupled with an elemental analyser (NA 1500 Series 2, Fisons Instruments). Calibrated N_2_ gas was used as the ^15^N/^14^N reference. Enrichments were expressed as atom percent (AP = ^15^N/(^14^N + ^15^N)) and atom percent excess (APE = AP – baseline ^15^N abundance of the sample) as described by Gausserès et al. [22]. Plasma concentrations of amino acids were measured by ion exchange chromatography, after deproteinisation with sulphosalicylic acid.

### Calculations

The amount of dietary N present in each ileal sample (N_meal_) was determined from the dilution of the isotopic marker (^15^N) as follows:
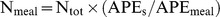
where N_tot_ is the amount of total N in the sample, and APE_s_ and APE_meal_ are ^15^N enrichment of the sample and the meal, respectively.

The level of endogenous N in samples was derived from the difference between N_tot_ and N_meal_. Apparent ileal digestibility (AID) was calculated from the cumulative amounts of total N recovered at the ileal level, corrected by the percentage of chromium recovery, using the following equation:
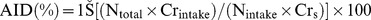



TID was calculated from the cumulated amounts of ^15^N recovered at the ileal level, corrected by the percentage of chromium recovery, using the following equation:
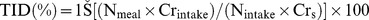
where N_intake_ is the amount of N ingested and Cr_intake_ and Cr_s_ are the amount of chromium in the diet and the digesta, respectively.

### Statistical analysis

Kinetics were analysed using the repeated option of the PROC MIXED procedure of SAS (SAS/STAT Users Guide®, Release 8.1; SAS Institute Inc., Cary, NC, 2000), with minipigs as the random effect and time, test meal, and time x test meal as factors. When a significant time x test meal interaction was found, the LSMEANS procedure was used to test differences at specific times, between and within test meals. The postprandial curve of indispensable amino acids (IAA) was characterised by baseline value (C_base_), zenith value (C_max_), time at which C_max_ was observed (tC_max_), and postprandial area under the curve (AUC; calculated by integrating the difference between C_base_ and the observed concentration, using the trapezoidal method). For cumulative fluxes at the ileum, AID, TID, and for the descriptive parameters of plasma amino acid curves, data were analysed by analysis of variance (ANOVA) with the GLM procedure of SAS, using a model with animal and test meal as independent variables. When test meal effect was significant (*P*<0.05), Duncan's test was used to compare means. Furthermore a post-hoc analysis was performed to determine the linear (L) and quadratic (Q) effects of cooking temperature (after the coefficients were adjusted for unequal spacing of temperature), or level of intake. All results are presented as means ± standard error of the mean (SEM).

## Results

^15^N labelling of calf muscles at slaughter was 0.44%. In exp. 1, juice losses during cooking were 16, 38, and 44%, at 60, 75, and 95°C, respectively. In exp.2, juice loss was 33% at a temperature of 75°C. Protein content (N ×6.25) of meat was 26, 32, and 35%, after cooking at 60, 75, and 95°C, respectively.

**Figure 1:**
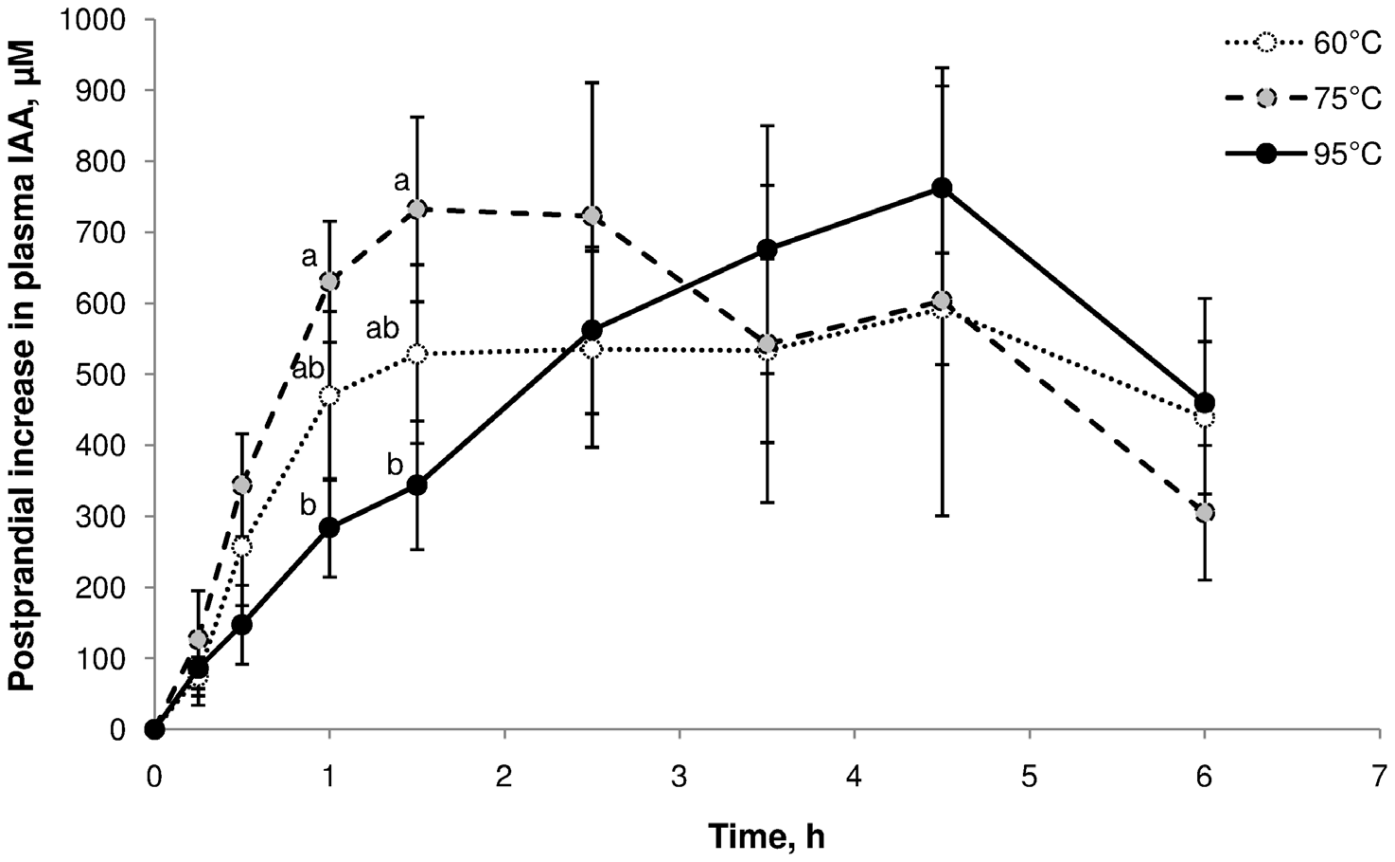
Effect of meat cooking temperature on the postprandial kinetics of plasma indispensable amino acids. Values are means ± SEM. Data were analysed by a mixed-model ANOVA with time as a repeated factor. Test meal effect over the first 3 h was significant (*P* = 0.0328). Means at a time without a common letter differ (*P*<0.05).

The effect of cooking temperature on the kinetics of plasma IAA concentration is presented in **Figure 1**. The change in concentration during the first 3 h after meal ingestion is mainly driven by the absorption of amino acids. Thus, the kinetics of plasma IAA concentration in this interval of time is a good index of the speed of digestion. Both the shape of the curve (**Figure 1**) and the AUC (**Table 1**) indicated an increase in the speed of digestion when meat cooking temperature was increased from 60 to 75°C, and a decrease in digestion speed when temperature was increased from 75 to 95°C. However, neither the maximal plasma IAA concentration, nor the AUC of the entire 6-h postprandial period were affected by cooking temperatures.

**Table 1:** Effect of meat cooking temperature on the postprandial kinetics of plasma indispensable amino acids^1^.

	Meat cooking temperature			Statistics^2^	
Item^3^	60°C	75°C	95°C	*P*	Effect^4^
C_base_, mM	975±59	1015±42	1008±25	0.817	
C_max_, mM	816±61	912±147	873±155	0.622	
tC_max_, min	162±35	165±62	290±24	0.113	
AUC_150 min_	517^b^±78	706^a^±106	420^b^±60	0.021	Q
AUC_360 min_	477±21	521±123	499±51	0.894	

1 Values are means ± SEM, n = 6.

2 Data were analysed by ANOVA with minipigs and meat cooking temperatures as main factors.

3 C_base_ = basal concentration in IAA; C_max_ = maximal increase in IAA concentration; tC_max_ = time at which C_max_ is observed; AUC = area under the curve (trapezoidal method) for the increase in IAA concentration over 150 or 360 min.

4 Quadratic (Q) effect of meat cooking temperature (*P*<0.05).

a,b Means within a row not sharing a common superscript differ (*P*<0.05).

**Figure 2:**
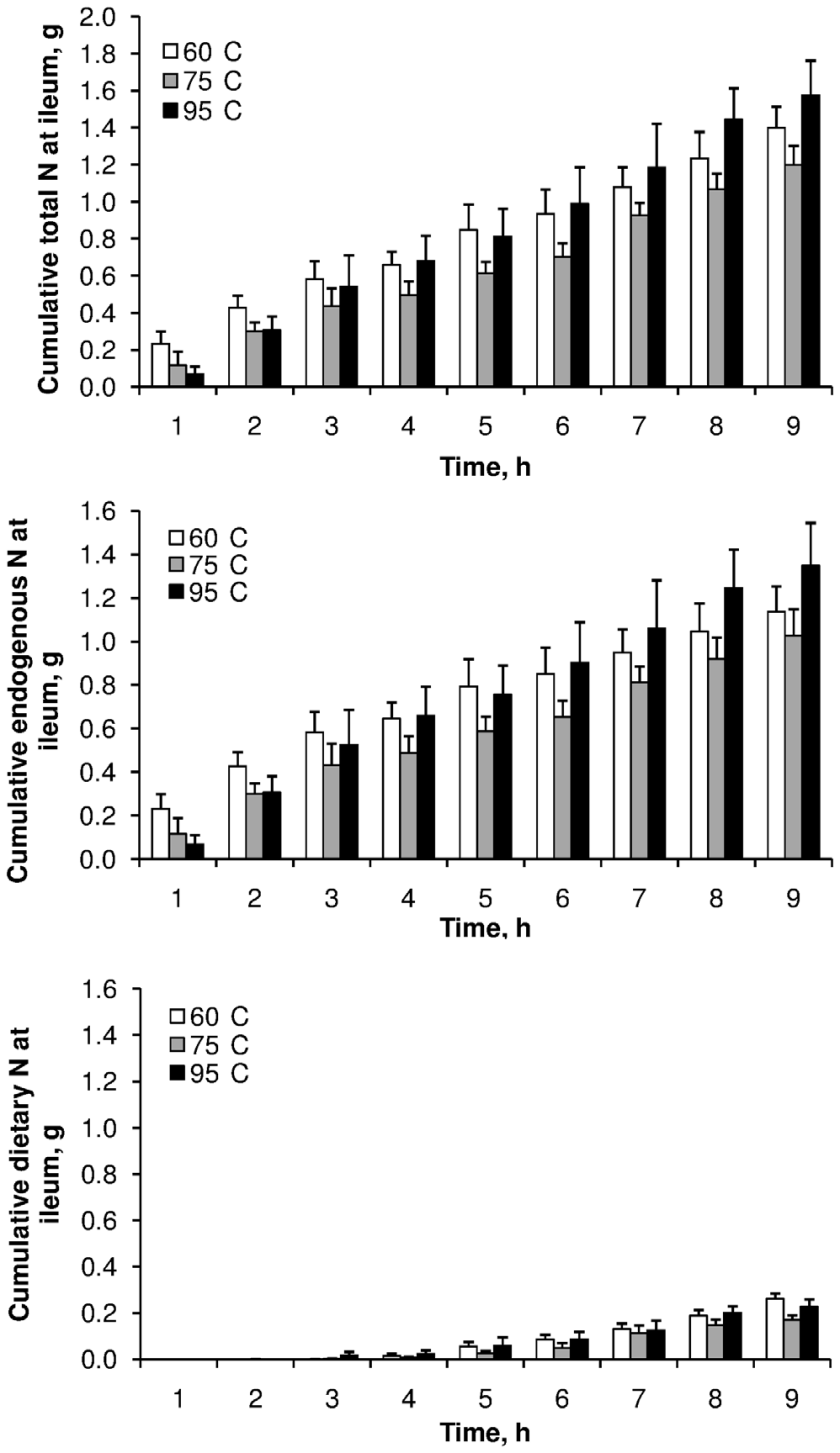
Effect of meat cooking temperature on the postprandial kinetics of ileal cumulative flux of nitrogen. Three different fluxes of nitrogen were determined: total, endogenous and dietary. Values are means ± SEM. Data were analysed by a mixed-model ANOVA with time as a repeated factor. No interaction was observed between cooking temperature and time after the meal (*P*>0.10).

**Figure 3:**
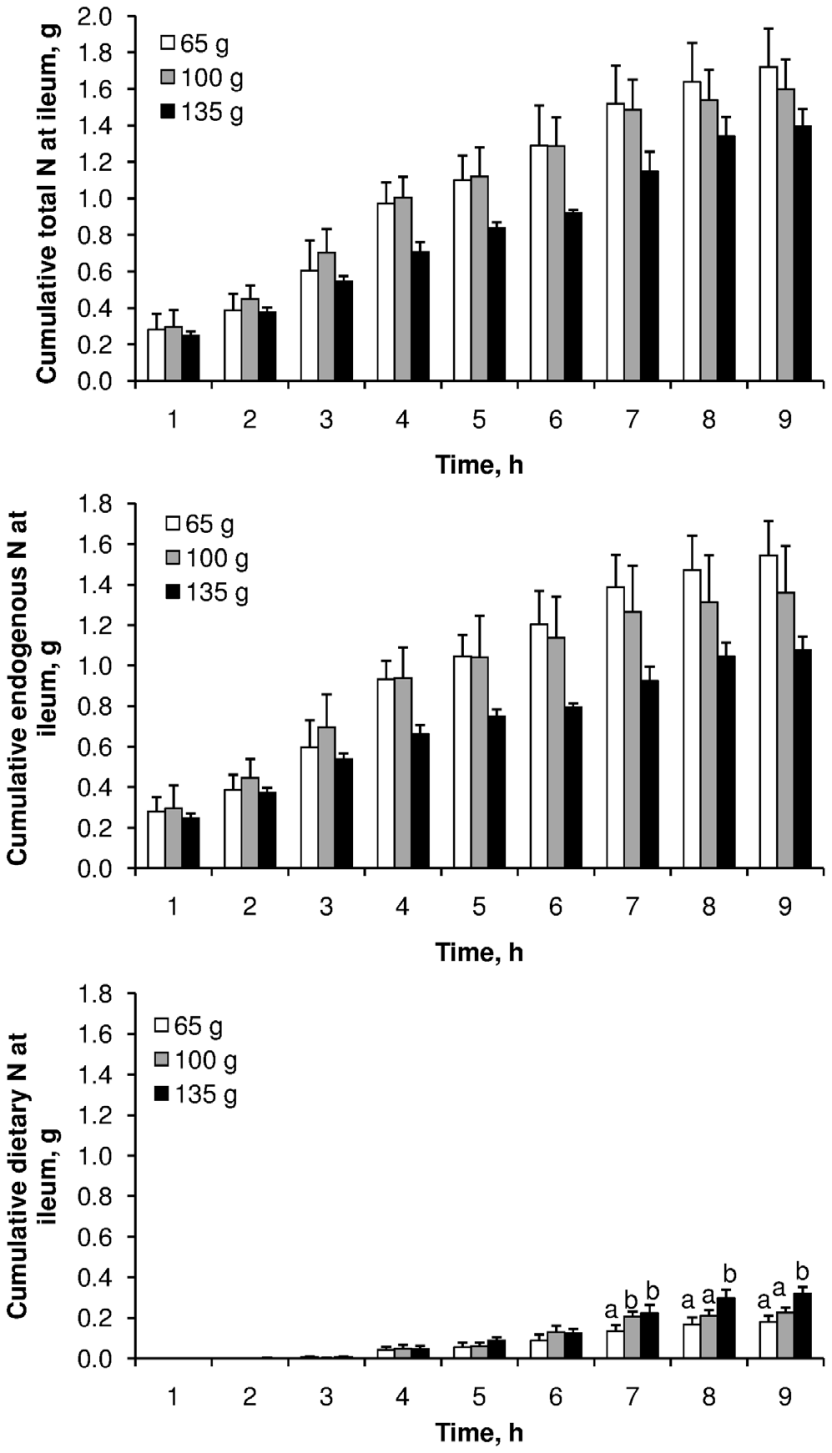
Effect of meat intake on the postprandial kinetics of ileal cumulative flux of nitrogen. Three different fluxes of nitrogen were determined: total, endogenous and dietary. Values are means ± SEM. Data were analysed by a mixed-model ANOVA with time as a repeated factor. Test meal x time interaction was significant for dietary nitrogen (*P* = 0.0314). Means at a time without a common letter differ (*P*<0.05).

**Figure 4:**
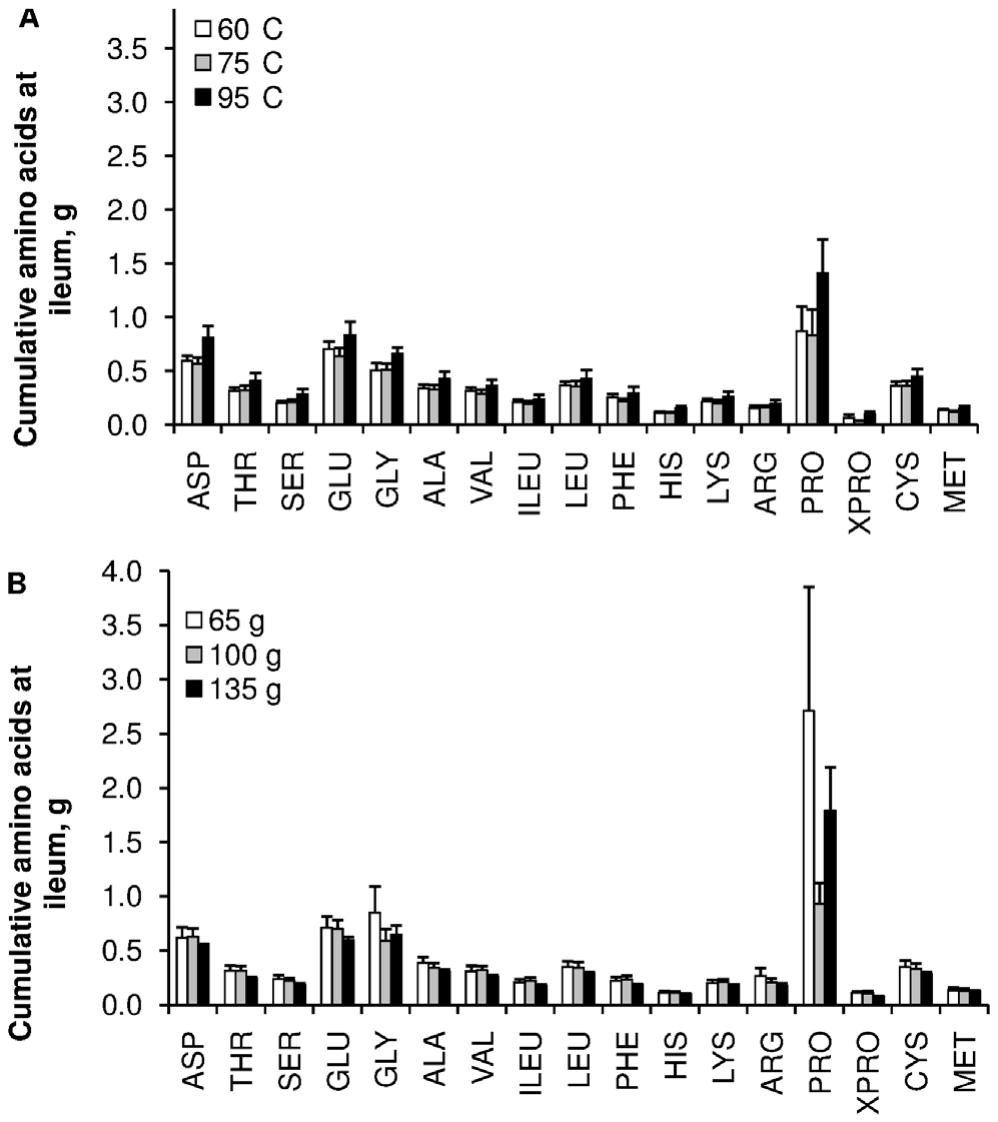
Effect of cooking temperature and level of intake, on the ileal cumulative flux of AA. Effect of meat cooking temperature [A] and amount of ingested meat [B]. Values are means ± SEM. Data were analysed by a mixed-model ANOVA with time as a repeated factor. No interaction was observed between cooking temperature or amount of ingested meat and time after the meal (*P*>0.10).

The cumulative curves of total, endogenous and dietary N, collected at the ileum after correction by chromium recovery, are presented in **Figure 2** and **Figure 3**. First appearance of labelled meat at the ileum was observed in the 3^rd^ hour following the meal. These curves were not significantly affected by meat cooking temperature; however, the cumulative dietary N measured between 7 and 9 h after the meal was greater when 135 g of meat was ingested than when 65 or 100 g were ingested. Total N flow to the ileum tended to be lower at the meat cooking temperature of 75°C (*P*<0.10), and consequently total N AID tended to be slightly greater at this temperature (*P*<0.10) (**Table 2**). Nevertheless, dietary N flow to the ileum and TID were not affected by meat cooking temperature (*P*>0.10). Similarly, the pattern of amino acids flowing at the ileum was not affected by meat cooking temperature (*P*>0.10) (**Figure 4A**). The increase in meat intake from 65 to 135 g did not affect total N flow to the ileum (*P*>0.10), consequently total N AID was largely increased (from 50 to 80%) (*P*<0.001) (**Table 3**). However, as dietary N flow increased (*P*<0.05), TID was not affected by the level of meat intake (*P*>0.10). This slight increase in dietary N flow did not affect the pattern of the amino acids flowing at the ileum (**Figure 4B**). On average, over exp. 1 and exp. 2, endogenous N amounted to 1.25 g over 9 h, accounting for 83% of the total N flowing at the ileum, and meat protein TID was 95.3±0.2%.

**Table 2:** Effect of meat cooking temperature on nitrogen flow to the ileum^1^.

	Meat cooking temperature			Statistics^2^
Item	60°C	75°C	95°C	*P*
Intake, g N	4.96	4.58	4.64	
Ileal digesta flow, g N/9 h				
Total N	1.40±0.12	1.20±0.09	1.58±0.19	0.094
Dietary N	0.26±0.02	0.17±0.02	0.23±0.03	0.132
Endogenous N	1.14±0.11	1.03±0.10	1.35±0.20	0.151
Apparent ileal digestibility, %	72.7±2.3	73.8±1.9	66.2±4.0	0.098
True ileal digestibility, %	94.7±0.5	96.3±0.4	95.1±0.7	0.299

1 Values are means ± SEM, n = 6.

2 Data were analysed by ANOVA with minipigs and meat cooking temperatures as main factors.

**Table 3:** Effect of the amount of ingested meat on nitrogen flow to the ileum^1^.

	Meat intake			Statistics^2^	
Item	65 g	100 g	135 g	*P*	Effect^3^
Intake, g N	3.42	5.13	6.83		
Ileal digesta flow, g N/9 h					
Total N	1.72±0.17	1.60±0.16	1.40±0.10	0.411	
Dietary N	0.18±0.03^b^	0.23±0.02^b^	0.32±0.03^a^	0.017	L
Endogenous N	1.54±0.17	1.36±0.19	1.08±0.07	0.176	
Apparent ileal digestibility, %	49.6±5.0^b^	68.7±3.2^a^	79.7±1.4^a^	0.001	L
True ileal digestibility, %	94.7±0.8	95.6±0.5	95.3±0.5	0.528	

1 Values are means ± SEM, n = 6.

2 Data were analysed by ANOVA with minipigs and amounts of ingested meat as main factors.

3 Linear (L) effect of meat intake (*P*<0.05).

a,b Means within a row not sharing a common superscript differ (*P*<0.05).

## Discussion

The concept of slow and fast proteins was established by Boirie et al. [23]. Postprandial utilisation of dietary amino acids by the body varies according to the speed of protein digestion and the physiology of the consumer. In young adults, it seems that slow proteins are more beneficial, avoiding the important oxidation of amino acids observed with fast proteins [24]. However, fast proteins are more efficient than slow proteins at improving postprandial protein anabolism in order to fight against the establishment of sarcopaenia in elderly individuals [1]. Similarly, the ingestion of fast proteins by sportsmen after exercise also seems to be more efficient than ingestion of slow proteins for sustaining muscle protein synthesis [25]. In this context, the ranking of food proteins according to their digestion rate is becoming an important challenge. The impact of food processing on this criterion has been clearly demonstrated using milk matrices [26]. A previous study showed that although meat is a source of fast proteins for the elderly [17], the speed of digestion can be affected by chewing efficiency. Even if the observed differences are tenuous by comparison with the differences observed for dairy proteins (caseins and whey proteins), the present study shows that speed of digestion can also be affected by meat cooking temperature.

Although the relationship is regularly questioned [15], red meat consumption has been suspected to be implicated in colorectal cancer development [27]. One of the hypotheses suggested to explain this link is that meat protein degradation in the colon leads to deleterious products such as sulphides or nitrosamines [28], [29]. The present study demonstrates a very high digestibility (about 95%) of meat proteins in the small intestine. This digestibility is not affected by meat preparation, and although the amount of meat protein entering the colon seemed to increase with the level of meat intake, it remained very low.

In the framework of human nutrition, the relevance of the pig as a model animal for protein digestion studies has been clearly established [30]. In the present study, veal was used as the model meat because the medium size of a calf offers a good compromise between the amount of meat produced and the cost of labelled amino acids. The cooking temperature of 60°C resulted in pink meat. The temperature of 75°C was the current cooking temperature for veal meat, and the temperature of 95°C represented boiled meat. The meat servings given to the minipigs correspond to servings of 180, 280 and 380 g of cooked meat for a man of 70 kg (1, 1.45, and 1.90 g protein/kg body weight), which corresponds to moderate-to-high amounts.

An *in vitro* approach has revealed that cooking temperature is one of the key determinants of digestion speed [19]. Relative to raw meat, the speed of digestion was increased at a cooking temperature of 70°C, and decreased at a cooking temperature above 100°C. This effect was explained by a progressive denaturation of proteins, which exposes cleavage sites to digestive enzymes, at low temperatures, and oxidation leading to protein aggregation, which hides cleavage sites, at high temperatures. Although *in vivo* regulatory factors (such as interactions with the other ingredients, enzyme secretions, gastric emptying, small intestinal tonus) are likely to contribute to the increase in plasma IAA observed in the present study, very similar variations to those recorded *in vitro* were observed, with the highest speed of digestion observed at a cooking temperature of 75°C. For the cooking temperatures of 60 and 75°C, the increase in plasma IAA was very rapid (within 15 min), and reached maximal values about 2.5 h after the beginning of the meal. These observations in minipigs are in agreement with previously reported data in humans with a meat cooking temperature of 65°C [17].

The determination of TID allows *in vivo* investigation of the effect of food processing on the bioavailability of amino acids derived from dietary protein degraded by gastrointestinal enzymes. In order to distinguish dietary residues from endogenous materials in ileal chyme, isotope dilution methods have been developed, which involve labelling either the food, or the animal. Both techniques have drawbacks [31]. The rapid recycling of ^15^N-labelled dietary proteins into endogenous secretions leads to underestimation of TID [32]. However, for animal labelling, controversies remain regarding the suitability of the amino acid used for labelling (generally ^15^N-leucine), and of the endogenous proteins used as a reference pool. The present study is the first to use labelled meat to determine the TID of its proteins. Meat was uniformly labelled by incorporation of a mixture of ^15^N-amino acids in order to avoid the uncertainties linked to the use of single amino acid labelling. The observed TID of meat proteins was high (95%), in agreement with that observed in humans for meat [18], and milk [4]. Although *in vitro* data suggested an increase in the digestibility of meat proteins with increased cooking temperatures [19], no such improvement in TID was observed in the present study. Heat treatments generally increase the Maillard reactions from basic amino acids, the production of disulphide bonds, and amino acid racemisation, which may potentially decrease digestibility [21], [33], [34]. This was not the case in the present study, where no effect was observed, even on individual amino acids such as lysine, arginine, and sulphur-containing amino acids. The meat proteins that escape small intestinal digestion seem to increase with the amount of ingested meat. As this increase was accompanied by a decrease in endogenous protein flow, a progressive overestimation of dietary N was likely to occur with the increase in the amount of ingested ^15^N, via the rapid recycling of N. Consequently, the TID probably slightly increased with the amount of ingested meat. Nevertheless, in agreement with these results, a study in humans with ileostomies showed that an increase of 60 g of ingested meat did not modify TID [18]. Thus, even with a high meat intake, very few meat proteins reached the colon. The main contributors to protein flow at this level of the gastrointestinal tract are of endogenous origin, and the profile of amino acids is not affected by the amount of ingested meat. Proline content at this level is high, but variable, probably in relation to individual variations in secretions of endogenous molecules, such as protein-rich peptides and mucines.

In conclusion, the digestibility of meat proteins in the small intestine is high, and whatever the cooking temperature and the level of intake, meat protein residues enter the colon at low levels. This study shows that the speed of protein digestion, a parameter of increasing interest in nutrition, can be modulated by meat preparation, a slower digestion being observed with high cooking temperature.
